# *Staphylococcus hominis* Infective Endocarditis Presenting with Embolic Splenic and Renal Infarcts and Spinal Discitis

**DOI:** 10.1155/2022/7183049

**Published:** 2022-05-14

**Authors:** David Vasconcellos, Bruce Weng, Patrick Wu, Gary Thompson, Made Sutjita

**Affiliations:** ^1^University of California, Riverside School of Medicine, Los Angeles, California, USA; ^2^Department of Internal Medicine, Riverside University Health System Medical Center, Moreno Valley, California, USA

## Abstract

*Staphylococcus hominis* (*S*. *hominis*) is a Gram-positive, coagulase-negative bacteria that occurs as a normal commensal organism on the skin and may rarely cause native valve endocarditis (NVE). We present a 62-year-old male with type 2 diabetes mellitus, coronary artery disease, and hypertension presenting with fever and abdominal pain. CT (computerized tomography) of the abdomen revealed splenic and renal infarcts; further imaging with MRI (magnetic resonance imaging) revealed enhancements consistent with discitis in T5-6 and L1-2. Three sets of blood cultures were positive for *S*. *hominis* sensitive to methicillin on antimicrobial susceptibility tests, and echocardiogram showed posterior mitral valve vegetation. The patient was initially treated with 10 weeks of nafcillin IV (intravenous) 2 g q4 hours. He had recurrent bouts of S. *hominis* bacteremia that was treated with IV vancomycin. His clinical course was complicated by new-onset atrial fibrillation with rapid ventricular response and congestive heart failure. Once bacteremia was cleared, his infective endocarditis was successfully definitively treated with mitral valve replacement and tricuspid repair.

## 1. Introduction

*Staphylococcus hominis* (*S. hominis*) is commonly recognized as a contaminant on blood cultures being a part of normal skin flora, but on rare occasion, may also cause native valve endocarditis (NVE) with embolic phenomena. Its predominance as a contaminant as well as less virulent properties than other more common infectious etiologies of endocarditis make *S. hominis* infection diagnostically challenging [1].

Infective endocarditis (IE) is caused by damage to the endocardium of the heart with subsequent colonization by an organism that adheres to the tissue creating a vegetation, more commonly occurring on damaged valves. With a reported incidence of 12.7 cases per 100,000 person-years [2], IE is rare, especially in populations without damaged heart valves or other structural heart conditions. Risk factors that may increase the incidence of IE include valvular insufficiency, degenerative cardiac lesions, congenital heart disease, prosthetic valves, and intravascular infection of cardiac devices. The most common cause of NVE in developed countries is *Staphylococcus aureus* (*S. aureus*), with one study showing up to 42% of NVE in nonnosocomial infections due to *S. aureus* [3]. Cultures isolating less common pathogens along with nonspecific symptoms of IE may lead to a delay in clinical diagnosis and treatment, increasing the risk for complications.

A feared complication of IE is septic embolization, which is due to partial dislodgement of the vegetation into the bloodstream causing spread to peripheral organs. Here, we present a case of native valve endocarditis due to *S. hominis* infection with embolic infarcts of the spleen and kidney and spinal discitis.

## 2. Case Presentation

A 62-year-old male with a history of type 2 diabetes mellitus, coronary artery disease, and hypertension who recently immigrated from Mexico presented to our hospital. He presented with complaints of acute lower abdominal pain with radiation to the left shoulder, back pain, and fever. He denied the history of previous abdominal surgery or intravenous (IV) drug use. The patient had a history of working in the farming industry, preparing feed for livestock; however, he denied having any direct contact with animals.

Significant physical exam findings included tenderness to palpation in the left upper quadrant of the abdomen, splenomegaly, and a blowing systolic murmur. Vital signs at initial examination are as follows: blood pressure 106/55 mmHg, heart rate 113 per minute, respiratory rate 24 per minute, body temperature 38.5°C, and oxygen saturation 94% on ambient air. Labs were remarkable for the following: hemoglobin 9.9 g/dL, sodium of 130 mmol/L, potassium of 3.5 mmol/L, creatinine 1.95 mg/dL, and AST of 57 U/L. Liver function tests were otherwise normal, and there was a normal peripheral white blood cell count. Urine cultures grew >100,000 colonies/mL coagulase-negative *Staphylococcus* (CoNS).

**Figure 1:**
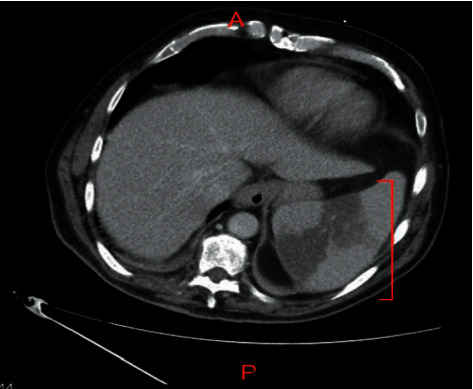
Axial CT abdomen and pelvis imaging showing an enlarged spleen with evidence of acute segmental infarction with well-defined hypodensities.

**Figure 2:**
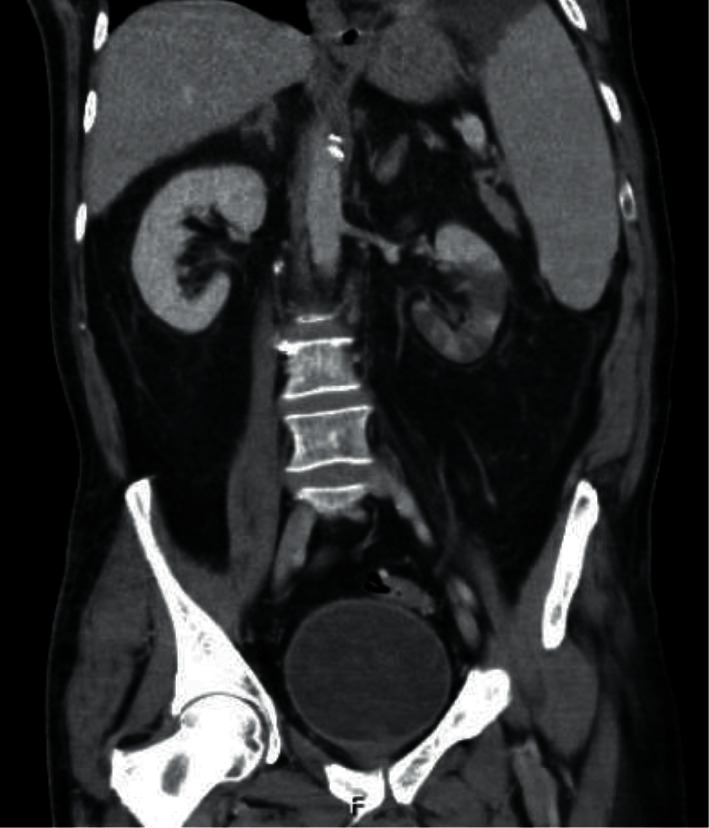
Coronal view of CT abdomen and pelvis showing numerous hypodensities of the interpolar region and lower pole of the left kidney consistent with infarctions.

Computerized tomography (CT) abdomen and pelvis with contrast showed numerous well-defined hypodensities in the spleen (Figure 1) as well as the left kidney consistent with infarction (Figure 2).

**Figure 3:**
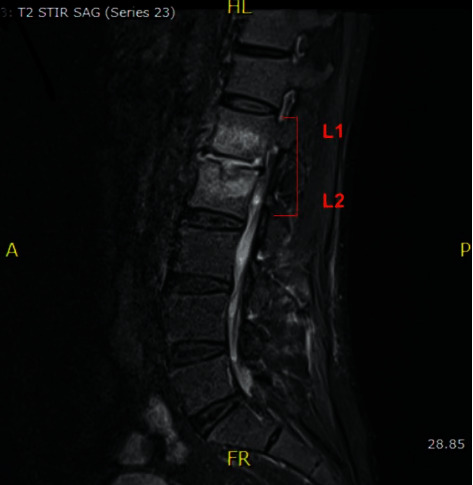
MRI of the spine with paraspinal soft tissue swelling and enhancement at the level of L1 and L2 as well as ventral epidural enhancement without rim-enhancing intraspinal or paraspinal fluid collections.

**Figure 4:**
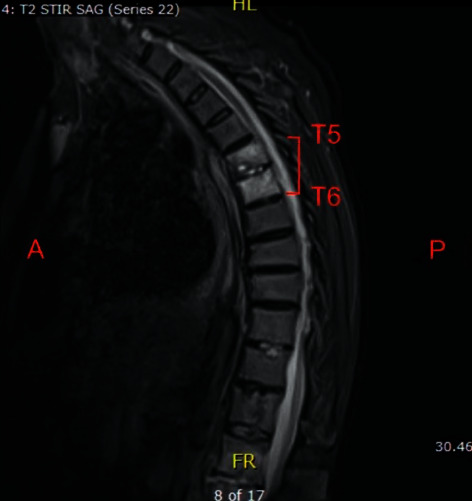
MRI thoracic spine with findings of discitis/osteomyelitis involving T5-6 with trace enhancement of the left anterior T5-6 epidural space.

Patient was initially treated empirically with IV cefepime and vancomycin for sepsis of unknown etiology. Magnetic resonance imaging (MRI) was ordered to investigate possible causes for this patient's back pain. MRI revealed findings of discitis and osteomyelitis of the lumbar (*L*) spine (Figure 3) and thoracic (*T*) spine (Figure 4).

**Figure 5:**
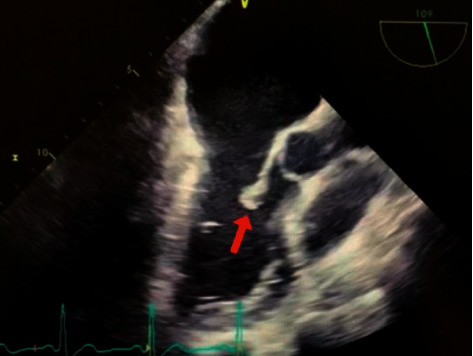
TEE findings noted mitral valve vegetations on the lateral posterior P2 segment (red arrow) and severe mitral regurgitation with systolic reversal of the right pulmonary vein.

Serial blood cultures revealed Gram-positive cocci in clusters which were later speciated as *S. hominis.* Transthoracic echocardiogram (TTE) revealed a moderate sized vegetation on the posterior mitral valve, and subsequent transesophageal echocardiogram (TEE) confirmed these findings along with severe mitral regurgitation. TEE otherwise revealed a normal left ventricular cavity, normal wall thickness, and ejection fraction of 60% with a similarly normal right ventricle (Figure 5). Serial blood cultures demonstrated persistent growth of *S. hominis* in multiple sets for two consecutive days, as well as a third blood culture 3 days after admission. Antibiotics were later changed to nafcillin once *S. hominis* susceptibilities resulted with susceptibility to methicillin. Due to the potential for atypical cause of infective endocarditis given the patient's employment working in the agricultural industry, further work-up included antibody titers for *Brucella*, *Coxiella*, and *Bartonella* spp. which were ultimately negative.

The patient's fever improved within 24 hours; he reported decreased abdominal pain and had clearance of blood cultures 4 days after initial presentation. He was then transferred to another hospital with cardiothoracic surgery services for mitral valve replacement on day 4 of admission. Patient received 6 weeks of intravenous nafcillin 2 g every 4 hours for 6 weeks. A lumbar spine biopsy was performed which was negative on culture for any organisms and on histopathology for any signs of malignancy. His regimen was extended by 4 weeks for a total of 10 weeks of IV nafcillin. Patient was seen for repeat bacteremia 4 months after initial presentation with *S*. *hominis* and *Staphylococcus epidermidis* (*S. epidermidis*) complicated by epididymitis, congestive heart failure, and new-onset atrial fibrillation with rapid ventricular response. Echocardiogram at this time revealed thickened tricuspid and mitral valves with no vegetation and an ejection fraction of 45–50%. *S*. *epidermidis* blood cultures were nonpersistent and considered a contaminant. He was treated with levofloxacin initially, which was then switched to ceftriaxone for epididymitis. He was again seen at 6 months after initial presentation for repeat bacteremia with *S. hominis* and acute heart failure exacerbation. The *S. hominis* bacteremia was treated with 8 weeks IV vancomycin, and following clearance of bacteremia, he underwent a successful mitral valve replacement and tricuspid valve repair.

## 3. Discussion

*S. hominis* is a CoNS that is a part of normal skin flora and rarely a cause of NVE, however, must still be considered in a patient presenting with fever, abdominal pain, and a new murmur. Of all causes of NVE, CoNS accounts for 5% of cases [4]. Of these cases of CoNS NVE, *S. epidermidis* is the most common causative pathogen. CoNS are emerging as a cause of NVE and have a rate of mortality similar to that of *S*. *aureus* infection, the most common cause of NVE [5, 6]. CoNS are normally considered less invasive as they are primarily skin commensal organisms not commonly causing serious infections. *Staphylococcus lugdunensis*, unlike other CoNS, can readily cause more severe and invasive infections and should be regarded as a dangerous opportunistic pathogen [7]. Clinicians must determine whether an isolated CoNS from blood culture is a contaminant or the cause of infection.

In patients with IE, prompt recognition of the causative pathogen is imperative. Pathogen-directed antibiotic treatment for IE should be started immediately as serious complications of systemic embolization, including, but not limited to, involvement of the central nervous system with ischemic stroke, intracranial hemorrhage, meningitis, and intracerebral abscess, may occur [8]. Although our patient had already presented with evidence of embolic phenomena, the timely diagnosis was essential in preventing further hemodynamic compromise, structural disruption of the valve, and further embolization. These cases may be difficult to treat as there may be complications of congestive heart failure and rhythm conduction abnormalities, as in our patient. Even in cases with aggressive medical and surgical treatment, mortality remains at 25% in cases of CoNS endocarditis [9]. Infective endocarditis due to CoNS is rare in native valves and is more commonly associated with infection in prosthetic valves; however, in a patient with persistently positive CoNS cultures and a clinical presentation clinically consistent with infection, IE must be evaluated.

## 4. Conclusion

Clinicians should have a high index for suspicion of CoNS endocarditis in a patient with serial positive blood cultures and embolic episodes though it is a rare cause of NVE. Antibiotic susceptibilities are required to guide prompt antibiotic therapy to decrease the risk of further complication in patients with extracardiac manifestations of IE.

## Data Availability

Due to the U. S. Health Insurance Portability and Accountability Act 1996 (HIPAA), the other data associated with the patient cannot be released.
